# Three Challenges in an Infant: Neuroblastoma Mimicking Infantile Hepatic Hemangioma, With Chylothorax and Rosettes in Pleural Fluid

**DOI:** 10.1155/crh/9948246

**Published:** 2025-07-03

**Authors:** Samin Alavi, Mitra Khalili, Maryam Kazemi Aghdam, Alireza Zamani, Tahereh Madani

**Affiliations:** ^1^Pediatric Congenital Hematologic Disorders Research Center, Research Institute for Children's Health, Shahid Beheshti University of Medical Sciences, Tehran, Iran; ^2^Department of Pediatric Radiology, Mofid Children's Hospital, Shahid Beheshti University of Medical Sciences, Tehran, Iran; ^3^Department of Pediatric Pathology, Mofid Children's Hospital, Shahid Beheshti University of Medical Sciences, Tehran, Iran; ^4^Payvand Clinical Laboratory, Tehran, Iran

**Keywords:** chylothorax, infantile hepatic hemangioma, liver tumor, neuroblastoma, pleural involvement, rosette cells

## Abstract

Infantile hepatic hemangioma (IHH) is rare, but the most common benign hepatic tumor in the first year of life. It has a characteristic course with perinatal presentation, increasing growth during the first year of life, and subsequent shrinkage of the vascular lesions. The authors report a 12-month-old male infant who presented with severe abdominal distension and respiratory distress while under workup for diffuse IHH since 2 months of age. In addition, the child's situation was complicated by two uncommon occurrences: bilateral chylothorax and the presence of neuroblasts and rosette cells in the pleural fluid. The detection of such cells in pleural fluid is extremely rare in pediatric neuroblastoma cases. This complex medical scenario highlights the challenges faced in diagnosing and managing rare pediatric conditions, emphasizing the need for careful monitoring and comprehensive diagnostic approaches in similar cases.

## 1. Introduction

Neuroblastoma (NBL) is the most common malignancy in infancy, originating from neural crest progenitor cells. It can occur wherever adjacent to the sympathetic nervous system, including the superior cervical, mediastinal, paraspinal, and celiac ganglia; however, the majority arises in the adrenal glands [1]. When evaluating diffuse liver lesions in infants, NBL, which is a common pediatric cancer typically seen around 18 months of age, must be considered in differential diagnosis. It is crucial to distinguish infantile hepatic hemangioma (IHH) from metastatic NBL due to their similar clinical presentation [2]. Accurate diagnosis is the key, especially when a prior diagnosis of IHH is made, as it affects treatment and prognosis significantly. Maintaining a broad differential diagnosis is essential to enhance the patient's outcome.

IHH is the most common hepatic vascular tumor in children. Liver tumors in infancy vary from vascular malformations to benign and malignant tumors including mesenchymal hamartoma, hepatoblastoma, and metastatic NBL [3]. The pathogenesis of IHH is not yet identified. It has been postulated that the origin of IHH is most probable congenital since they demonstrate embryonic features [4].

Here, we report an infant with metastatic NBL to liver, para-aortic, retrocrural, and mediastinal paravertebral regions at 12 months of age, who was diagnosed with IHH since 2 months of age. There is not any evidence in the literature to indicate IHH as a predisposing factor for NBL. The proposal that hepatic mass lesions in the present case could have been metastatic NBL since early infancy or there could be a comorbidity occurring almost simultaneously will be further discussed. The present case also had two other unique presentations; the occurrence of bilateral chylothorax in the course of the disease and observation of tight clusters of small round cells and rosette cells in pleural fluid that makes the case extremely rare in children with NBL.

## 2. Case Report

A 12-month-old male infant was admitted to the pediatric oncology department with progressively worsening abdominal distention and mild respiratory distress, initially presenting with diffuse hepatic mass lesions identified at 2 months of age. Initial ultrasonography revealed significant hepatomegaly with multiple hypoechoic masses throughout the liver, characterized by high vascularity, the largest measuring 60 × 45 × 38 mm in the right liver lobe. Subsequent MRI confirmed multiple large hepatic masses with enhancement suggestive of IHH. Other abdominopelvic organs, including adrenals, appeared normal. The infant showed no symptoms and had stable ultrasound results until his first birthday, when he experienced sudden abdominal distension necessitating further evaluation.

**Figure 1:**
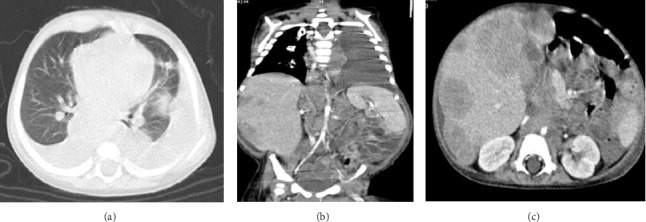
(a) Chest CT scan showed bilateral moderate pleural effusion. A large anterior mediastinal mass (45 × 30 mm) and left supraclavicular lymphadenopathies were observed. ((b) and (c)) abdominal-pelvic MRI revealed massive enlargement of the liver with numerous mass lesions, multiple conglomerated different-sized masses in retroperitoneum and lesser sac, associated with lymphadenopathies around aorta and its branches, superior mesenteric and renal arteries with extension to retrocrura and posterior mediastinum. The adrenals were spared.

On physical examination, the baby exhibited some degrees of respiratory distress. The liver border was extended up to 6 cm below costal margin to the iliac crest. Routine laboratory investigations were within normal limits except for mild anemia and increased serum LDH to more than 10,000 IU/dL. Ultrasonography of abdomen at admission showed heterogeneous enlarged liver with multiple hypoechoic masses with the largest measuring 67 × 35 mm in left liver lobe along with multiple large paravertebral masses in upper abdomen, adjacent to celiac trunk with internal calcification encasing aorta and its branches near to the aortic bifurcation (organ of zuckerkandl). Right-sided pleural effusion was also evident for which the patient underwent chest CT, which showed bilateral moderate pleural effusion and multiple paravertebral masses from the level of T3–L4. Likewise, large anterior mediastinal mass (45 × 30 mm) and left supraclavicular lymphadenopathies were observed. Abdominal-pelvic MRI revealed massive enlargement of the liver with numerous mass lesions, multiple conglomerate different-sized masses in retroperitoneum and lesser sac, associated with lymphadenopathies around aorta and its branches, superior mesenteric, and renal arteries with extension to retrocrura and posterior mediastinum; however, the adrenals were spared (Figure 1).

**Figure 2:**
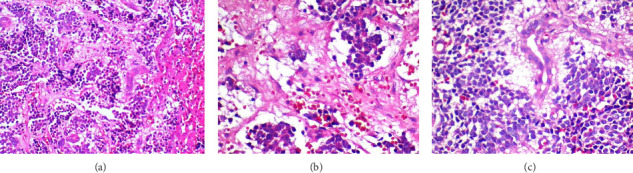
(a) Poorly differentiated neuroblastoma with fibrillary background infiltrated hepatic tissue (H&E ×200), (b) malignant tumor nests with fibrillary background (H&E ×400), (c) poorly differentiated neuroblastoma with hyperchromatic and some vesicular nuclei in the periphery of a biliary duct (H&E ×400).

**Figure 3:**
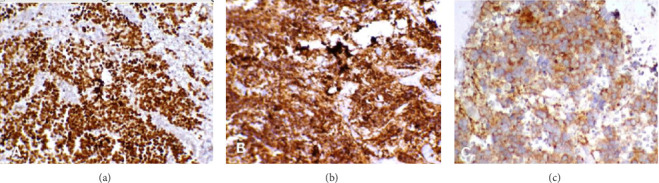
(a) Nuclear staining for PHOX2B IHC marker (×200), (b) cytoplasmic staining for synaptophysin IHC marker (×200), (c), cytoplasmic staining for chromogranin IHC marker (×200).

The 24-h urinary catecholamine metabolites (VMA and HVA) were highly increased. The patient underwent laparotomy through which biopsy from the tumor of celiac axis and liver lesions were performed. The pathology was consistent with poorly differentiated NBL with liver metastasis (Figure 2). Immunohistochemistry of tissue sample was positive for PHOX2B, synaptophysin, and chromogranin (Figure 3). The FISH method detected MYCN amplification with MYCN:NM1 ratio of 6.76. There was an average of 13.5 copies of MYCN per cell.

**Figure 4:**
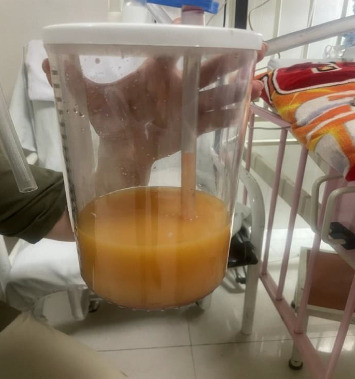
Chylothorax of the patient with neuroblastoma.

**Figure 5:**
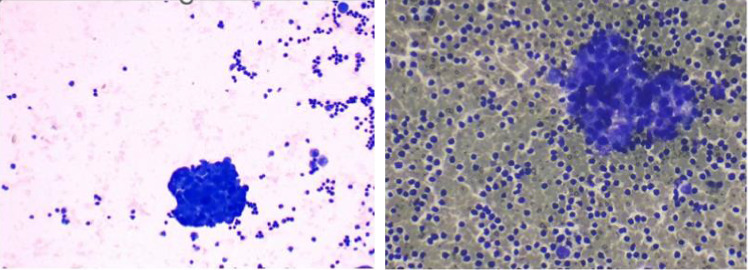
Tight clusters of small round cells (rosette cells) in the pleural fluid.

To relieve respiratory distress, chest tube drainage was inserted on the right side in which about 350 mL of milky white-colored fluid was drained (chylothorax) on the first day (Figure 4). It was exudative with total protein 3.2 g%, sugar 87 mg%, LDH 1120 U/L, and triglyceride 1003 mg/dL. The cytomorphology of the pleural fluid was as follows: WBC 680/mm^3^, with lymphocyte 85%, neutrophil 7%, a few mesothelial cells, and a population of large cells with high N/C ratio and conspicuous nucleoli arranged as tight clusters of small round cells (rosette cells) (Figure 5). Immunophenotyping of the pleural fluid revealed the presence of an abnormal CD 56 bright, CD 45 negative population in favor of pleural involvement with NBL. The drainage of chylous gradually decreased so that the chest tube was removed after about 2 weeks. Chemotherapy with the protocol assigned for high-risk NBL according to COG (NCT03126916) was started, while fat-free diet, MCT oil, and octreotide infusion were prescribed for the management of chylothorax. The infant had a partial response after three courses with dramatic reduction in abdominal distension and gradual disappearance of pleural effusion on both sides. At the time of writing this manuscript, the patient is undergoing the BIT protocol of bevacizumab, irinotecan, and temozolomide in preparation for MIBG therapy with significant reduction in size of the liver lesions. The protocol aims to achieve a very good partial response, paving the way for potential stem cell transplantation.

## 3. Discussion

We presented a case of NBL in an infant with severe hepatomegaly, progressive abdominal distension, and respiratory distress with past history of multiple hepatic hypoechoic lesions since 2 months of age. Imaging studies revealed heterogeneously enhancing mass lesions in liver and para-aortic area with extension to the posterior mediastinum and retrocrural space, producing paravertebral mass lesions compressing mediastinal structures including thoracic duct, and bilateral severe pleural effusion in which the histopathology was consistent with poorly differentiated NBL.

A population-based registry-driven study described maternal anemia, neonatal hemolytic disease, low Apgar score at 1 min, and neonatal respiratory distress as risk factors for the development of NBL in infancy [5]. It is also well recognized that patients with diseases of neural crest origin, such as congenital central hypoventilation syndrome and Hirschsprung disease, are at increased risk for neuroblastic tumors, compared with general population [6]. The PHOX2B gene, which was the first-known gene predisposing to NBL, was identified in 2004. Subsequently, mutations in PHOX2B gene have been identified in the neural crest developmental disorders and familial NBL [7, 8]. Since IHH has not been recognized as a risk factor for the development of NBL and given that IHHs are most commonly detected incidentally during postnatal ultrasonography, it is assumed that the large tumoral lesions in the liver, starting at 2 months of age in the present case, were indicative of metastases of a NBL. There is a case report of metastatic NBL misdiagnosed as infantile hemangioma in a 4‐month‐old child [2]. According to the literature, three patterns of focal, multifocal, and diffuse IHH have been identified in infancy. These tumors commonly have an increasing pattern of growth in early infancy, reach their maximal size by 5 months of age, and then display a regressive course [9] whereas in the present case, there were large hepatic lesions which did not involute but grew increasingly, resulting in severe abdominal distension.

As is the case, pulmonary involvement in NBL has been reported very rarely. The European Neuroblastoma Study Group (ENSG) has documented pulmonary disease in 35 out of 746 patients with stage IV NBL at diagnosis during a 10-year period. Pleural effusion, pleural infiltration, and lung lesions were detected in 5, 18, and 13 patients, respectively [10]. In the same way, the frequency of pleural effusion in NBL is much rarer, not exactly determined.

In a study from St. Jude Children's Research Hospital between 1991 and 2005, 31 out of 295 (10.5%) patients (3 months–20 years, median: 2 years), with NBL having pleural effusion at the time of presentation. The primary disease in this cohort was abdomen in 26 and mediastinum in 5 patients. Cytologic analysis of pleural fluid showed malignant cells in seven specimens [11]. The difference in the survival rate of the patients with or without malignant pleural effusion was not reported to be statistically significant.

In the present case, the pleural fluid had two unique features: first, it was a chylous fluid confirmed by its color and triglyceride content and second, it was positive with malignant cells and their clusters as rosette formation. The oldest report of pleural effusion due to NBL is a 14-year-old girl who had massive pleural effusion on the left, which was positive for groups of small round tumor cells [12]. A four-month-old male baby has also been reported who showed evidence of milky white pleural effusion due to a large thoracic paravertebral mass. Cytology of this case was also suggestive of round tumoral cells, most probably NBL [13].

There are very limited reports of childhood malignancies associated with chylothorax, which include lymphoma, NBL, germ cell tumor, Kaposi sarcoma, and Wilms tumor [14–16]. Our patient had large supraclavicular and retrocrural lymphadenopathies that could have contributed to the blockage of the thoracic duct. The authors of two other cases of NBL in early infancy with chylothorax have also suggested the compressive effects of the mediastinal adenopathy as the main culprit for developing chylothorax [15, 16]. Interestingly, a full-term neonate who required intubation and ventilatory support at birth was confirmed to have bilateral chylothorax. She underwent thoracotomy for mediastinal NBL 13 days after birth [17]. There is a report of a neonate in the literature with congenital chylous ascites, which resolved with complete resection of a retroperitoneal favorable histology NBL [18].

The chylothorax in NBL needs to be managed similarly to the other malignant etiologies. The first goal would be to relieve respiratory symptoms by drainage of the pleural fluid. In cases with persistent respiratory compromise and voluminous fluid or if there is a high possibility of recurrence, a chest tube should be inserted for continuous drainage of the pleural space. In the present case, insertion of the chest tube and continuous infusion of octreotide along with initiation of systemic chemotherapy resulted in a gradual reduction of the bilateral chylothorax.

## 4. Conclusion

When encountering diffuse hepatic lesions in early infancy, it is crucial to evaluate the potential for hepatic metastasis of NBL. NBL, primarily a pediatric malignancy, rarely involves the lungs. Nonetheless, vigilant monitoring of chest images is recommended for any NBL patient to assess unexpected pulmonary involvement. In cases where pleural fluid is detected, it is essential to perform aspiration and subsequent analysis. The identification of malignant cell clusters within the pleural fluid signifies advanced disease and is associated with a poor prognosis in pediatric patients with NBL. This underscores the importance of thorough diagnostic evaluations and vigilant monitoring to inform prognosis and guide treatment strategies effectively.

## Data Availability

The data that support the findings of this study are available from the corresponding author upon reasonable request.

## References

[1] Maris J. M. (2010). Recent Advances in Neuroblastoma. *New England Journal of Medicine*.

[2] Karkoska K., Ricci K., VandenHeuvel K. (2021). Metastatic Neuroblastoma Masquerading as Infantile Hemangioma in a 4‐month‐old Child. *Pediatric Blood and Cancer*.

[3] Gnarra M., Behr G., Kitajewski A. (2016). History of the Infantile Hepatic Hemangioma: From Imaging to Generating a Differential Diagnosis. *World Journal of Clinical Pediatrics*.

[4] Blei F., Walter J., Orlow S. J., Marchuk D. A. (1998). Familial Segregation of Hemangiomas and Vascular Malformations as an Autosomal Dominant Trait. *Archives of Dermatology*.

[5] Bluhm E., McNeil D. E., Cnattingius S., Gridley G., El Ghormli L., Fraumeni Jr J. F. (2008). Prenatal and Perinatal Risk Factors for Neuroblastoma. *International Journal of Cancer*.

[6] Rohrer T., Trachsel D., Engelcke G., Hammer J. (2002). Congenital Central Hypoventilation Syndrome Associated with Hirschsprung’s Disease and Neuroblastoma: Case of Multiple Neurocristopathies. *Pediatric Pulmonology*.

[7] Trochet D., Bourdeaut F., Janoueix-Lerosey I. (2004). Germline Mutations of the Paired–like Homeobox 2B (PHOX2B) Gene in Neuroblastoma. *The American Journal of Human Genetics*.

[8] Bourdeaut F., Trochet D., Janoueix-Lerosey I. (2005). Germline Mutations of the Paired-like Homeobox 2B (PHOX2B) Gene in Neuroblastoma. *Cancer letters*.

[9] Krowchuk D. P., Frieden I. J., Mancini A. J. (2019). Clinical Practice Guideline for the Management of Infantile Hemangiomas. *Pediatrics*.

[10] Cowie F., Corbett R., Pinkerton C. R. (1997). Lung Involvement in Neuroblastoma: Incidence and Characteristics. *Medical and Pediatric Oncology: The Official Journal of SIOP—International Society of Pediatric Oncology Societé Internationale d’Oncologie Pédiatrique.*.

[11] Gupta H., Conrad J., Khoury J. D. (2007). Significance of Pleural Effusion in Neuroblastoma. *Pediatric Blood and Cancer*.

[12] Ghosh P. (1968). Pleural Effusion Due to Neuroblastoma. *British Journal of Diseases of the Chest*.

[13] Anand S., Agrawal A. (2012). Neuroblastoma Presenting as Chylothorax. *Pediatric Oncall Journal*.

[14] Tutor J. D. (2014). Chylothorax in Infants and Children. *Pediatrics*.

[15] Seshachalam A., Nandennavar M., Laxmi L., Sagar T. (2010). Nontraumatic Chylothorax in a Case of Neuroblastoma. *Indian Journal of Cancer*.

[16] Totadri S., Trehan A., Bhattacharya A., Bansal D., Attri S. V., Srinivasan R. (2017). Chylothorax in Children with Cancer: a Milky Predicament. *Indian Journal of Cancer*.

[17] Easa D., Balaraman V., Ash K., Thompson B., Boychuk R. (1991). Congenital Chylothorax and Mediastinal Neuroblastoma. *Journal of Pediatric Surgery*.

[18] Pai V. B., Benator R., Torres B. (2017). Refractory Chylous Ascites Secondary to Neuroblastoma. *Fetal and Pediatric Pathology*.

